# The Monkeypox Fear Scale: development and initial validation in a Peruvian sample

**DOI:** 10.1186/s40359-022-00997-0

**Published:** 2022-11-28

**Authors:** Tomás Caycho-Rodríguez, Lindsey W. Vilca, Carlos Carbajal-León, Miguel Gallegos, Mario Reyes-Bossio, Martin Noe-Grijalva, Mariel Delgado-Campusano, Águeda Muñoz-del-Carpio-Toia

**Affiliations:** 1 Facultad de Ciencias de la Salud, Universidad Privada del Norte, Av. Alfredo Mendiola, Los Olivos, Lima, 6062 Peru; 2 South American Center for Education and Research in Public Health, Universidad Norbert Wiener, Lima, Peru; 3 Departamento de Psicología, Facultad de Ciencias de la Salud, Universidad Católica del Maule, Talca, Chile; 4 Pontificia Universidade Católica de Minas Gerais, Belo Horizonte, Brasil; 5 Facultad de Psicología, Universidad Nacional de Rosario, Rosario, Argentina; 6 Consejo Nacional de Investigaciones Científicas y Técnicas, Buenos Aires, Argentina; 7 Facultad de Psicología, Universidad Peruana de Ciencias Aplicadas, Lima, Peru; 8 Escuela de Psicología, Universidad César Vallejo, Trujillo, Peru; 9 Escuela de Postgrado, Escuela de Medicina Humana, Universidad Católica de Santa María, Arequipa, Peru; 10 Universidad de FLACSO, Buenos Aires, Argentina; 11 Instituto de Ética Clínica Francisco Vallés, Universidad Europea, Madrid, Spain

**Keywords:** Scale, Monkeypox, Fear, Validation, Peru

## Abstract

**Background:**

Fear is one of the basic emotions generated during periods of infectious diseases. Therefore, the aim of this study was to develop and validate a scale that assesses monkeypox fear, the Monkeypox Fear Scale (MFS).

**Methods:**

A total of 451 Peruvians participated (61% women and 39% men), with a mean age of 28.31 years (SD = 9.72). based on procedures from classical test theory (CTT) and item response theory (IRT). Classical Test Theory (CTT) and Item Response Theory (IRT) procedures were used.

**Results:**

The results showed that MFS has a two-factor structure related to emotional and physiological fear factors (χ2 = 41.87; df = 12; *p* < .001; CFI = .99; TLI = .99; RMSEA = .074 [IC90% .051–.100]). In addition, the physiological and emotional factors showed good reliability. Measurement invariance analysis showed that the factor structure of the MFS is strictly invariant between male and female groups. Finally, the discrimination and difficulty parameters of the items show adequacy. In addition, the scale seems to be more accurate in measuring high levels of fear of monkeypox.

**Conclusion:**

The MFS has adequate psychometric evidence to assess fear of monkeypox in the Peruvian population. These findings may guide future studies related to the consequences of monkeypox on mental health.

## Introduction

The World Health Organization (WHO) has declared Monkeypox as a Public Health Emergency of International Concern (PHEIC) on July 23, 2022, due to the progressive increase of infections in different parts of the world [1]. According to the latest WHO situation report, as of September 14, a total of 59,147 laboratory-confirmed cases and 22 deaths were reported in 103 countries worldwide [2]. These cases were reported in countries where Monkeypox was not endemic, marking the first time that cases have been detected without direct links to Africa. Therefore, for WHO, the occurrence of a single confirmed case represents an outbreak. In Peru, on May 19, 2022, an epidemiological alert was issued for the possible presence of Monkeypox in the country. The purpose of this is for the different public and private health organizations and institutions to identify, notify and investigate compatible cases of Monkeypox [3]. The first case in Peru was confirmed on June 27 and since then there has been a considerable increase in the number of cases [4]. As of September 16, 2091 cases of Monkeypox were reported nationwide in Peru [5].

The above situation has generated an international alert for governments and national health systems, due to the implications on the demand for medical care. In the meantime, some studies have begun to investigate attitudes, knowledge and preventive practices regarding monkeypox. The data indicate that awareness of monkeypox disease was quite low among health professionals and the general population, at least until the end of May [6, 7]. Both studies were conducted before the declaration of PHEIC by the WHO, suggesting that with the increase in cases and greater public dissemination of information, concern on the part of the general population will increase, as well as a deeper understanding of the disease.

Current interest in monkeypox is focused on the control and treatment of the disease, as well as the presence of an effective vaccine [8, 9]. However, the psychosocial aspects associated with an infectious disease must be considered. The resurgence and spread of monkeypox represents a risk to physical and mental health, particularly in non-endemic countries such as Peru. The emergence of the COVID-19 pandemic has heightened the fear of a new infectious epidemic [10, 11]. Previous outbreaks of monkeypox were accompanied by fear and concerns about stigmatization and social exclusion of infected patients, survivors and family members [12]. It has been suggested that the current fear of monkeypox may be due to its similarity to the dreaded and eradicated smallpox virus, case fatality rates of up to 11%; more frequent person-to-person transmission; lack of clarity about the source and mode of transmission of the virus; faster geographic spread; lack of data on the efficacy of available antivirals in treatment, among other factors [13, 14]. It should be noted that fear is associated with the speed, means of transmission, morbidity and mortality of a disease [15]. Higher levels of fear would cause people to have less clarity and rationality in reacting to monkeypox.

While countries should take various actions to reduce the rate of monkeypox transmission, they should also focus their efforts on individual fears associated with infectious diseases. The lack of attention to the fear of monkeypox, and other mental health problems [16], is the absence of a suitable instrument to measure it. Developing a brief measure of monkeypox fear with evidence of validity and reliability is timely and important. Therefore, this study aimed to develop and validate a scale that assesses monkeypox fear, the Monkeypox Fear Scale (MFS), based on procedures from classical test theory (CTT) and item response theory (IRT). CTT considers the test, in this case the MFS, as the unit of analysis, while IRT considers the items as the unit of analysis. IRT assumes that the psychometric properties of a test are independent of the sample and provides item parameters. This allowed us to identify the most discriminating items that can measure fear of monkeypox more reliably. Specifically, we evaluated the evidence of validity based on content and internal structure, reliability, item characteristics based on the IRT, and measurement invariance (MI) according to gender. MI allows to be certain that the same construct can be measured equivalently among different groups. On a practical level, this would allow differences to be interpreted as true and not biased by instrumental problems [17, 18]. MFS can be useful in providing important information on the fear of monkeypox to assist in the formulation of public health initiatives, such as programs to manage fear of the disease in the general population.

## Method

### Participants and procedure

The sample included 451 participants (61% female and 39% male), with a mean age of 28.31 years (SD = 9.72). Most participants were single (79.2%), with completed (31.3%) or incomplete (40.1%) college education, living in urban areas (91.4%), diagnosed with COVID-19 (63.2%), and vaccinated against COVID-19 (98.7%). Likewise, 97.6% do not live with vulnerable people and 91.8% do not suffer from any chronic disease. Finally, 99.6% have not been infected with monkeypox; while 97.6% have no relatives or friends infected with monkeypox. Details of the demographic characteristics of the participants are shown in Table 1.

**Table 1:** Characteristics of the sample under study

	n	%
Age		
Gender		
Female	275	61%
Male	176	39%
Marital Status		
Married	45	10%
Single	357	79.2%
Cohabitant	34	7.5%
Divorced	11	2.4%
Widowed	4	.9%
Academic degree		
Incomplete elementary school	1	.2%
Primary school complete	0	0%
Incomplete high school	5	1.1%
High school complete	74	16.4%
Technical studies incomplete	8	1.8%
Technical studies complete	41	9.1%
Incomplete University	181	40.1%
University complete	141	31.3%
Lives in		
Urban	412	91.4%
Rural	39	8.6%
Had COVID-19		
Yes	285	63.2%
No	166	36.8%
Vaccinated against COVID-19		
Yes	445	98.7%
No	6	1.3%
Contagious with monkeypox		
Yes	2	.4%
No	449	99.6%
Relatives or friends infected with monkeypox		
Yes	11	2.4%
No	440	97.6%
Vulnerable person at home		
Yes	278	61.6%
No	173	38.4%
Suffering from any chronic illness		
Yes	37	8.2%
No	414	91.8%

Participants were selected through a snowball convenience sampling. Inclusion criteria were: (1) Peruvian nationality; (2) being of legal age; and (3) being able to respond to online surveys. The determination of the number of participants followed the recommendations for factor analysis based on the CTT and IRT model-based analyses, where 300 to 375 participants is adequate to obtain significant results [19–21]. Data was collected through an online survey between August 15 and September 10, 2020. The online survey was shared via social media, email, and WhatsApp.

### Instrument

Monkeypox Fear Scale (MFS). The MFS was designed based on the Spanish version of the Fear of COVID-19 Scale (FCV-19S) validated in different Latin American countries [22] and evaluates symptoms of fear of the monkeypox. Based on this, fear would be expressed in emotional and physiological reactions (two-factor model). In this sense, to design the MFS, the items of the FCV-19S in Spanish were adapted to the monkeypox context. For this, the term "COVID-19″ was changed to "monkeypox" in each item. For example, the item "I feel uncomfortable to think about Coronavirus." was changed to " I feel uncomfortable thinking about monkeypox”. This same procedure was used to develop other scales that measure emotions or cognitions associated with infectious diseases, such as concern about the contagiousness of VIDOC-19 [23] and conspiracy beliefs about COVID-19 vaccines [24]. Thus, the MFS is made up of seven items, which have five Likert-type response options ranging from 1 = strongly disagree to 5 = strongly agree.

The MFS items are presented in English and Spanish below:

1. I am very afraid of monkeypox (Tengo mucho miedo a la viruela del mono).
2. I feel uncomfortable thinking about monkeypox (Me incomoda pensar sobre la viruela del mono).
3. My hands become clammy when I think about monkeypox (Mis manos se vuelven húmedas cuando pienso en la viruela del mono).
4. I am afraid of losing my life to monkeypox (Tengo miedo de perder la vida por la viruela del mono).
5. When I see news and stories about monkeypox on social media, I get nervous or anxious (Cuando veo noticias e historias sobre la viruela del mono en las redes sociales, me pongo nervioso o ansioso).
6. I can't sleep because I worry about having monkeypox (No puedo dormir porque me preocupa tener la viruela del mono).
7. My heart races when I think about getting monkeypox (Mi corazón se acelera cuando pienso en contraer la viruela del mono).

### Data analysis

First, item statistics (mean [M], standard deviation [SD], skewness [g1] and kurtosis [g2]) were calculated using SPSS 22.0 for Windows. Secondly, the evidence of content validity was evaluated based on the criteria of clarity, coherence, and relevance of the MFS items by a set of 6 expert judges (psychologists, psychiatrists, and epidemiologists) contacted through their e-mails. Relevance is the degree to which the item is important and should be included to assess the construct fear of monkeypox; coherence is the degree of relationship between the item and the measured construct; while clarity is the degree to which the item is clear and understandable.

All criteria are scored from 0 (not at all relevant/coherent/clear) to 3 (totally relevant/coherent/clear). The quantification of the degree of clarity, coherence and relevance of the items was performed with Aiken's V coefficient [25] and its 95% confidence intervals (95% CI) [26]. The V values vary between 0 and 1, where values greater than .70 express a positive assessment of the items at the sample level, and values of the lower limit (Li) of the 95% CI greater than .59 are adequate at the population level.

Then, a Confirmatory Factor Analysis (CFA) was performed using the estimator *Diagonally Weighted Least Squares with Mean and Variance corrected* (WLSMV) since the items are ordinal in nature [27]. The fit of the models was evaluated with the RMSEA, SRMR, CFI and TLI indices. RMSEA and SRMR values less than .08 are considered acceptable [28]; while CFI and TLI values above .95 were considered adequate [29]. The reliability of the scale was estimated by calculating the omega coefficient [30]. Values of ꞷ > .80 are adequate [31].

Subsequently, for the IRT-based analyses, a Graduated Response Model was used (GRM) [32]. Specifically, an extension of the 2-parameter logistic model (2-PLM) was used for ordinal polytomous items [33]. Two types of parameters were estimated for each item: discrimination (a) and difficulty (b). Due to the presence of five response categories in each item, four estimates of difficulty were reported, one for each threshold. These estimates indicated the level of the latent variable at which a person has a 50% probability of scoring equal to or greater than a specific response category. The information curves for the items (CII) and the information curve for the test (CIT) were also calculated.

The MI of the MFS according to gender of the participants was performed based on a sequence of restrictive hierarchical variance models (configural invariance, metric invariance, scalar invariance, and strict invariance). Comparison of the sequence of models was first performed with a formal statistical test, which is the chi-square difference (Δχ2), where nonsignificant values (*p* > .05) indicate MI between groups. Then, a modeling strategy was used based on differences in CFI (ΔCFI), where values less than < .010 suggest MI of the model between groups [34].

Statistical analyses were performed with the "lavaan" package [35] for AFC, the "semTools" package [36] for MI and the "ltm" package for GRM [37] In all cases, the RStudio environment [38] for R [39] was used.

### Ethical considerations

The project was approved by the Institutional Committee for the Protection of Human Subjects in Research (CIPSHI) of the University of Puerto Rico (No. 2223-006). All subjects participated anonymously and voluntarily. In addition, they gave their informed consent online at the beginning of the survey. In addition, the study also followed the ethical principles of the Declaration of Helsinki [40] (and the American Psychological Association [41].

## Results

### Validity based on the content of the items

Table 2 reports that all seven M items received favorable evaluations of their clarity, relevance, and consistency (V > .70). Similarly, the lower bounds of the confidence intervals of all SFM items satisfy the population-level criterion (Li > .59).

**Table 2:** Aiken's V for assessing the clarity, coherence, and relevance of MFS items

Item	Clarity (n = 6)				Coherence (n = 6)				Relevance (n = 6)			
	M	DE	V	IC95%	M	DE	V	IC95%	M	DE	V	IC95%
Item 1	2.83	.41	.94	.78–.99	3.00	.00	1.00	.87–1.00	2.50	.84	.83	.65–.93
Item 2	2.67	.52	.89	.71–.96	2.50	.55	.83	.65–.93	2.83	.41	.94	.78–.99
Item 3	2.50	.55	.83	.65–.93	3.00	.00	1.00	.87–1.00	3.00	.00	1.00	.87–1.00
Item 4	2.17	.41	.72	.53–.86	2.33	.82	.78	.59–.90	2.33	.82	.78	.59–.90
Item 5	2.67	.82	.89	.71–.96	3.00	.00	1.00	.87–1.00	2.67	.52	.89	.71–.96
Item 6	2.67	.82	.89	.71–.96	2.67	.52	.89	.71–.96	2.83	.41	.94	.78–.99
Item 7	2.60	.52	.87	.69-.95	2.60	.70	.87	.69–.95	2.90	.32	.97	.82–.99

### Descriptive analysis

The average, standard deviation, skewness, kurtosis and polychoric correlation matrix of the MFS items are reported in Table 3. It is observed that item 1 (I am very afraid of monkeypox) had the highest average score (M = 2.88); whereas item 6 (I can't sleep because I am worried about having monkeypox) had the lowest average score (*M* = 1.76). The polychoric correlation matrix of the items indicated the presence of moderate and high correlation coefficients. Likewise, all items presented adequate skewness and kurtosis indices in the sample (> ± 1.5).

**Table 3:** Descriptive analysis of items and polychoric correlation matrix

Items	Polychoric correlation matrix						
	1	2	3	4	5	6	7
1. Am I very afraid of monkeypox?	1						
2. Does it make me uncomfortable to think about monkeypox?	.62	1					
3. Do my hands get wet when I think about monkeypox?	.53	.50	1				
4. Am I afraid of losing my life to monkeypox?	.62	.46	.66	1			
5. When I see news and stories about monkeypox on social media, do I get nervous or anxious?	.67	.60	.71	.69	1		
6. I can't sleep because I am worried about having monkeypox?	.53	.53	.81	.66	.77	1	
7.- Does my heart race when I think about getting monkeypox?	.58	.53	.79	.69	.80	.89	1
Mean	2.88	2.80	1.82	2.23	2.27	1.76	1.95
Standard Deviation	1.19	1.22	1.08	1.22	1.21	1.02	1.18
Skewness	.04	.17	1.26	.61	.60	1.24	1.07
Kurtosis	− .87	− .88	.80	− .75	− .66	.84	.13

*M* = Mean; *SD* = Standard Deviation; *g1* = Skewness; *g2* = Kurtosis

### Validity based on internal structure

**Fig. 1:**
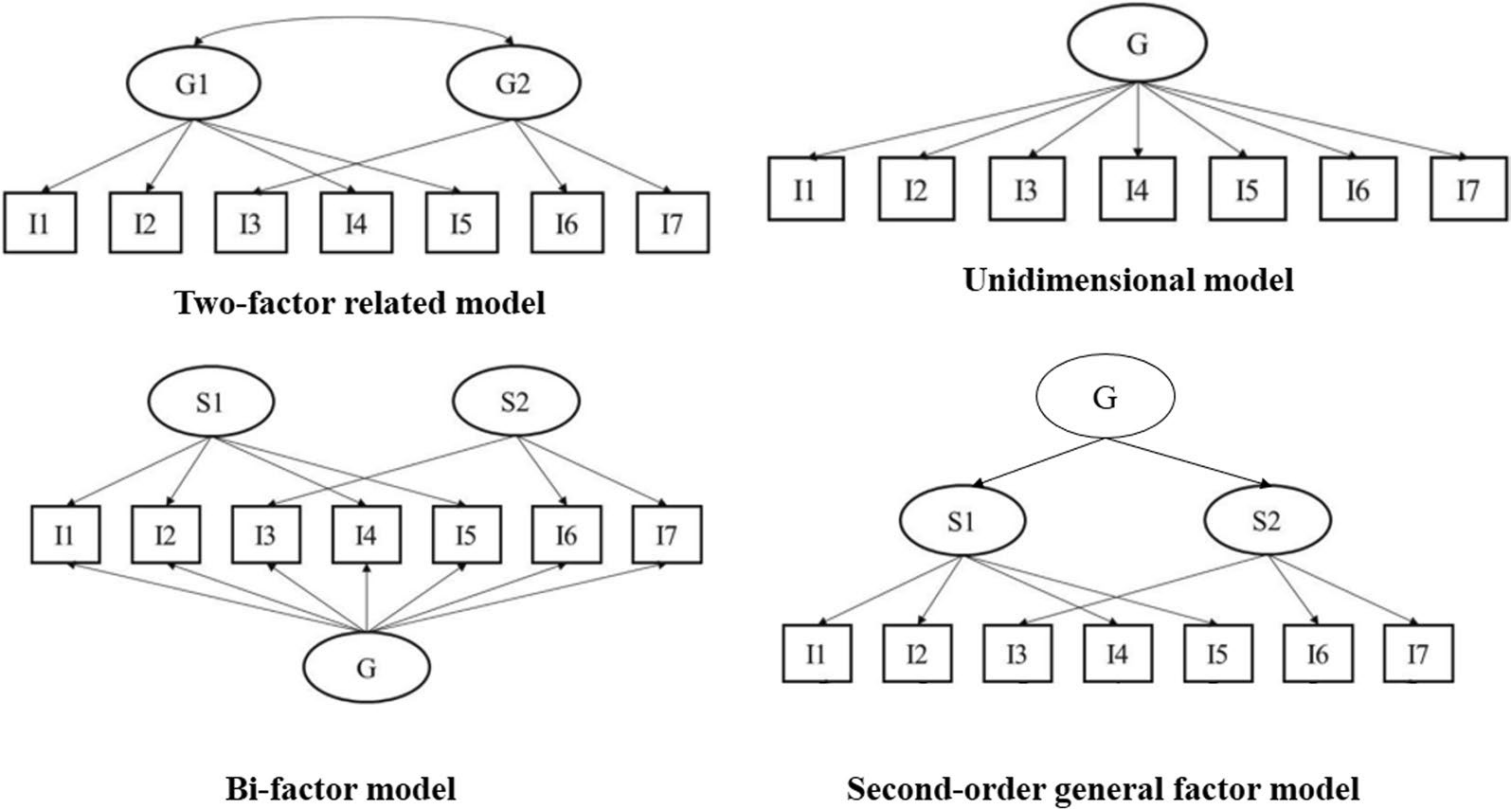
Competing models

Table 4 shows that the two-factor related model, which is based on the FCV-19S from which the MFS is derived, has adequate fit indices in the total sample of participants (χ2 = 41.87; df = 12; *p* < .001; CFI = .99; TLI = .99; RMSEA = .074 [IC90% .051–.100]). However, as these two dimensions present a high level of correlation (.91), other competing models were evaluated: unidimensional model, bi-factor model and second-order general factor model (see models in Fig. 1). The bi-factor and second-order general factor models presented estimation and convergence problems. Regarding the unidimensional model, it was found that it does not fit the data (χ2 = 105.42; df = 13; *p* < .001; CFI = .99; TLI = .98; RMSEA = .126 [IC90% .104–.148]). In contrast, the two-factor related model has adequate fit indices in the group of men (χ2 = 21.09; df = 12; *p* = .049; CFI = .99; TLI = .99; RMSEA = .066 [IC90% .004–.111]) and women (χ2 = 26.73; df = 12; *p* = .008; CFI = .99; TLI = .99; RMSEA = .067 [IC90% .032–.101]). It can also be seen that in the total sample and in the specific groups, the factorial weight of the latent variable with each of its items are high and significant (see Table 5). Therefore, the two-factor related model was used in the following psychometric analyses.

**Table 4:** Two-factor related model fit indices and sex-invariant models

Models	χ^2^	df	*p*	SRMR	TLI	CFI	RMSEA [CI 90%]	Δχ^2^	Δdf	*p*	ΔCFI
Total sample											
Two-factor related model	41.87	12	.000	.025	.99	.996	.074 [.051–.100]	‒	‒	‒	‒
One-dimensional model	105.42	13	.000	.042	.98	.988	.126 [.104–.148]	‒	‒	‒	‒
Two-factor model a	‒	‒	‒	‒	‒	‒	‒	‒	‒	‒	‒
General factor model a	‒	‒	‒	‒	‒	‒	‒	‒	‒	‒	‒
According to gender								‒	‒	‒	‒
Male	21.09	12	.049	.029	.99	.997	.066 [.004–.111]	‒	‒	‒	‒
Female	26.73	12	.008	.023	.99	.997	.067 [.032–.101]	‒	‒	‒	‒
Configural	33.83	24	.088	.021	.98	.988	.043 [.000–.074]	‒	‒	‒	‒
Metric	33.84	29	.245	.029	.99	.994	.027 [.000–.060]	4.93	5	.423	.006
Scalar	39.89	34	.225	.031	.99	.993	.028 [.000–.058]	6.00	5	.305	− .001
Strict	44.85	41	.313	.037	.99	.995	.020 [.000–.051]	6.09	7	.529	.002

χ2 = Chi square; df = degrees of freedom; SRMR: Standardized Root Mean Square Residual; TLI = Tucker-Lewis Index; CFI = Comparative Fit Index; RMSEA = Root Mean Square Error of Approximation; Δχ2 = Differences in Chi square; Δdf = Differences in degrees of freedom; ΔCFI = Change in Comparative Fix Index. ^a^ = A solution has NOT been found

**Table 5:** Standardized factor weights of the items and reliability of the scale according to sex, age, and total sample

Items	Total Sample (n = 451)		Male (n = 176)		Female (n = 275)	
	Factor 1	Factor 2	Factor 1	Factor 2	Factor 1	Factor 2
	λ (error)	λ (error)	λ (error)	λ (error)	λ (error)	λ (error)
3	.86 (.26)		.86 (.26)		.86 (.27)	
6	.93 (.13)		.95 (.11)		.94 (.12)	
7	.95 (.10)		.96 (.08)		.94 (.11)	
1		.71 (.49)		.76 (.43)		.68 (.53)
2		.63 (.60)		.66 (.57)		.67 (.63)
4		.79 (.37)		.78 (.39)		.80 (.37)
5		.92 (.16)		.88 (.22)		.93 (.13)
Reliability						
α	.89	.83	.90	.81	.89	.83
ω	.91	.79	.92	.81	.91	.78

λ = factor loadings; Factor 1 = Physiological dimension; Factor 2 = Emotional dimension

### Scale reliability

Table 5 shows that the physiological (ω = .91) and affective (ω = .79) dimensions of the MFS present adequate reliability in the total sample of participants. Similar results are found in the group of males: physiological (ω = .92) and emotional (ω = .81) dimension; and females: physiological (ω = .91) and affective (ω = .78) dimension.

### Factorial invariance by age

Table 4 shows that the factor structure of the scale exhibits evidence of being strictly invariant between male and female groups in the sequence of invariance models proposed: metric (ΔCFI = .006), scalar (ΔCFI = − .001) and strict (ΔCFI = .002) invariance.

### Item response theory model: graded response model (GRM)

Two graded response models (GRM) were fitted, specifically a 2PLM model for each dimension of the MFS. Table 6 shows that all the discrimination parameters of the physiological and emotional dimension items are above the value of 1, generally considered as good discrimination [33]. Regarding the difficulty parameters, in both dimensions, all threshold estimators increased monotonically, as expected.

**Table 6:** Discrimination and difficulty parameters for the items of each dimension

Dimensions	Item	a	b_1_	b_2_	b_3_	b_4_
Physiological	M3	3.49	− .38	.48	1.19	1.81
	M6	3.69	− .30	.46	1.43	2.11
	M7	3.49	− .52	.21	.93	1.54
Emotional	M1	2.45	− 1.24	− .31	.61	1.63
	M2	1.79	− 1.27	− .21	.79	1.77
	M4	2.21	− .37	.37	1.15	2.15
	M5	3.27	− .40	.35	1.06	1.87

a = discrimination parameters; b = difficulty parameters

**Fig. 2:**
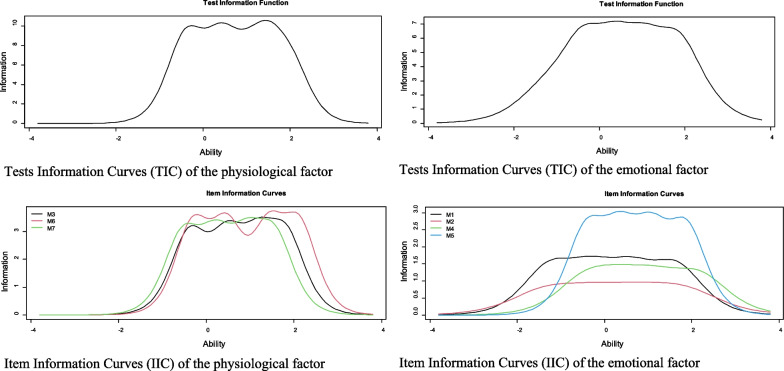
Item and test information curves for the scale

Figure 2 shows the Information Curves for the items and dimensions (IIC and ICT respectively). Regarding the physiological dimension, the IIC shows that item 6 is the most accurate in assessing the latent trait. In addition, the TIC shows that the factor is more reliable (accurate) in the range of the scale between − 1 and 2. Regarding the emotional dimension, the IIC shows that item 5 is the most accurate in assessing the latent trait. In addition, the ICT shows that the factor is more reliable (accurate) in the range of the scale between − 1 and 2.5.

## Discussion

A better understanding of the impact of monkeypox on physical and mental health is a major concern today. Therefore, this study aimed to develop and validate the Monkeypox Fear Scale (MFS), a recently developed measure to evaluate the fear of monkeypox in a Peruvian sample. For this purpose, classical psychometric methods, such as the CFA, and modern methods, i.e., IRT analysis, were used.

First, the evaluation of the content of the seven MFS items indicated that they are sufficiently relevant, coherent, and clear to adequately represent the construct fear of monkeypox, both at the sample level (V ≥ .70) and at the population level (Li > .59). That is, the content of the seven items is adequate to be applicable to the Peruvian sample. The CFA compared various factor models (two-factor related, unifactor, two-factor, and second-order general factor) and indicated that the two-factor related model presented an adequate fit to the data. These factors comprised items expressing emotional and physiological reactions to monkeypox fear. Furthermore, the reliability of this two-factor related model is adequate, both in the total sample and the subsamples of men and women, indicating that the MFS is an accurate measure of emotional and physiological symptoms of fear. The presence of emotional and physiological symptoms of monkeypox fear has also been observed during the COVID-19 pandemic in Latin American countries [22, 42–44]. This result gives grounds to argue that, during infectious diseases of global relevance, people feel threatened and tend to answer questions and express their fear in a similar way. Having a two-factor model allows researchers and health professionals to differentiate between the fear of monkeypox and its associated emotional and physiological symptoms.

Further, the MI assessment indicated that the MFS is a measure that is strictly invariant between groups of men and women. Specifically, the presence of configurational invariance indicates that, the basic factor structure of the MFS is invariant between males and females. Therefore, both groups conceptualize the construct fear of monkeypox, as measured by the MFS, in a similar way. Similarly, metric invariance would indicate that the factor loadings of the MFS are similar, which would indicate that both gender groups respond to the items in the same way. This would make it possible to make comparisons between male and female groups. On the other hand, it has also been observed that the intercepts of the items are also invariant between genders (scalar invariance). This would allow comparisons of latent means between the groups between genders. Finally, strict invariance provided support for comparisons of correlations between fear of monkeypox and other variables between the groups [45]. These findings are even more important if one takes into consideration that fear is affected by inherent gender characteristics [46]. In this regard, studies during infectious diseases, such as COVID-19, have suggested that they have had a greater psychological impact on women compared to men [47].

CTT-based analyses are useful for understanding the psychometric properties of the MFS as a totality, where the results will depend on the sample. However, IRT methods consider items as the unit of analysis, where the measurement accuracy of an item will depend on the latent trait of an individual. The IRT analysis indicated that all items have adequate discrimination parameters. This indicates that all MFS items significantly discriminate between those with low, medium, and high levels of fear of monkeypox. Specifically, item 6 (I can't sleep because I worry about having monkeypox) is the most discriminative and accurate in assessing the physiological dimension of fear. Recent studies have indicated the presence of sleep disturbances during periods of infectious disease [48]. It is suggested that it is difficult to be certain whether the symptoms of fear generate sleep problems or whether the sleep problems produce the fear, so it is more likely that the relationship is bidirectional [49]. Furthermore, item 5 (When I see news and stories about monkeypox on social networks, I get nervous or anxious) is the most discriminative and accurate item for assessing the emotional dimension of fear. The COVID-19 pandemic has provided evidence that increased exposure to news of diagnosed cases and/or deaths from infectious diseases, such as monkeypox, increases fear and other mental health symptoms [50, 51]. Finally, the findings suggest that an individual must have higher latent traits (in our case, greater fear of monkeypox) to respond to the higher response options in the MFS. In this way, the seven items of the MFS have good characteristics to evaluate the fear of monkeypox in the Peruvian population.

It has been mentioned that there is a limited number of validated instruments to assess mental health aspects associated with monkeypox. Therefore, the development of the MFS is important. However, the present study has limitations that should be considered when interpreting the results. First, the use of non-probabilistic sampling techniques generates a selection bias, which prevents us from observing how representative the findings are for the entire Peruvian population. This has resulted in most participants being women, single people, with university studies, either complete or incomplete, and living in urban areas. Future studies should work with representative samples based on probability sampling techniques. Second, the use of an online survey to collect information limits that only people with Internet access can be part of the sample. In addition, it has been suggested that answering questions online may result in the presence of anxiety symptoms or other negative emotional reactions [52]. This leaves out people without internet access and who are not familiar with online surveys. Nevertheless, online surveys also make it possible to reach a larger number of people and reduce data loss. Third, the use of a self-report measure to collect data on monkeypox fear may generate social desirability bias or other method biases. Fourth, the research only provided evidence for validity based on internal structure, item characteristics, and MI, but not on convergent and discriminant validity with other variables associated with monkeypox fear. Therefore, future research should establish strong evidence for the convergent and discriminant validity of the MFS.

Despite the limitations, the study also has important implications. First, research conducted during the monkeypox public health emergency would benefit from the inclusion of a measure of fear of monkeypox, either as an outcome variable or as an explanatory variable associated with mental health. Second, having a validated measure such as the MFS would allow us to identify levels of fear of monkeypox among different groups of men and women. This could be useful for locating groups at potential risk for mental health problems associated with monkeypox. Similarly, MFS could be useful to decision makers and health professionals in developing and evaluating mental health programs for people who may experience fearful symptoms during the monkeypox public health emergency.

In conclusion, the study makes available to the scientific community a psychometrically promising measure to assess symptoms of fear during the monkeypox public health emergency and identify those individuals who may be in need of mental health care. Recently, epidemiological studies have used short measures to assess the degree of fear of monkeypox in the general Peruvian population as a measure for early diagnosis. Still, further research on the psychometric evidence and use of SFM is needed to lead to new empirical and theoretical findings on the emotional and physiological responses to monkeypox fear. In addition, researchers from other Spanish-speaking countries could use the MFS in other to verify the psychometric findings and create norms for the instrument. This is important, considering that there are cultural differences in the Spanish-speaking world, which may be significant in the experience of fear of illness.

## Data Availability

The datasets generated during and/or analyzed during the current study are available from the corresponding author on reasonable request.
